# Perforated duodenal diverticulum successfully treated with a combination of surgical drainage and endoscopic nasobiliary and nasopancreatic drainage: a case report

**DOI:** 10.1186/s40792-020-00891-0

**Published:** 2020-06-08

**Authors:** Ayako Shimada, Koji Fujita, Minoru Kitago, Shunsuke Ichisaka, Keiichi Ishikawa, Hiroyuki Kikunaga, Koichiro Kumai, Hiroshi Miura

**Affiliations:** 1 Department of Surgery, Hino Municipal Hospital, 4-3-1 Tamadaira, Hino, Tokyo, 191-0062 Japan; 2 Department of Hepato-Biliary-Pancreatic and Gastrointestinal Surgery, International University of Health and Welfare Narita Hospital, Narita, Japan; 3 Department of Surgery, Keio University School of Medicine, Shinjuku, Japan; 4 Department of Radiology, Hino Municipal Hospital, Hino, Japan

**Keywords:** Perforation, Duodenal diverticulum, Endoscopic treatment

## Abstract

**Background:**

Perforation of a duodenal diverticulum is a rare complication that may become fatal with a delay in appropriate treatment. However, the optimal treatment for perforated duodenal diverticulum remains controversial, ranging from conservative therapy to surgery including pancreatoduodenectomy.

**Case presentation:**

The patient was a 60-year-old woman with no particular medical history who visited our hospital with chief complaints of continuous fever and right dorsal pain. Upon arrival, she had tenderness in the right upper quadrant of the abdomen. Laboratory data showed the elevation of inflammatory markers. Computed tomography revealed free air with abscess formation around the duodenum, which was diagnosed as duodenal perforation with abdominal abscess. We decided on emergent surgery, and we identified the perforation site on the dorsal side of the second portion of the duodenum intraoperatively. However, the inflammation around the perforation site was severe, and it was difficult to perform primary closure or dissection of the perforated diverticulum. Therefore, we finished surgery by placing four indwelling intra-abdominal tubes. Since postoperative day (POD) 1, the elevation of inflammation markers appeared to be uncontrollable, owing to the leakage of bile and pancreatic juice. We decided to perform endoscopic retrograde cholangiopancreatography on POD 2, and inserted endoscopic nasobiliary drainage and nasopancreatic drainage tubes. The patient showed a good postoperative course and was discharged on POD 57.

**Conclusions:**

Endoscopic nasobiliary and nasopancreatic drainage in combination with surgical drainage may be an effective treatment for perforated duodenal diverticulum.

## Background

Duodenal diverticulum is a relatively common disease and usually asymptomatic unless complications occur [1]. Perforation of a duodenal diverticulum is a rare complication that may become fatal with a delay in diagnosis owing to bile and pancreatic juice leakage [2–7]. However, the most appropriate treatment for perforated duodenal diverticulum remains disputable, which ranges from conservative therapy to surgery, including pancreatoduodenectomy. In addition to these treatments, we consider that endoscopic treatment may play a large role to control the leakage of bile and pancreatic juice [8, 9]. Here, we experienced a case with a perforated duodenal diverticulum, which we successfully treated by surgery followed by endoscopic drainage of the bile and pancreatic ducts.

## Case presentation

**Fig. 1:**
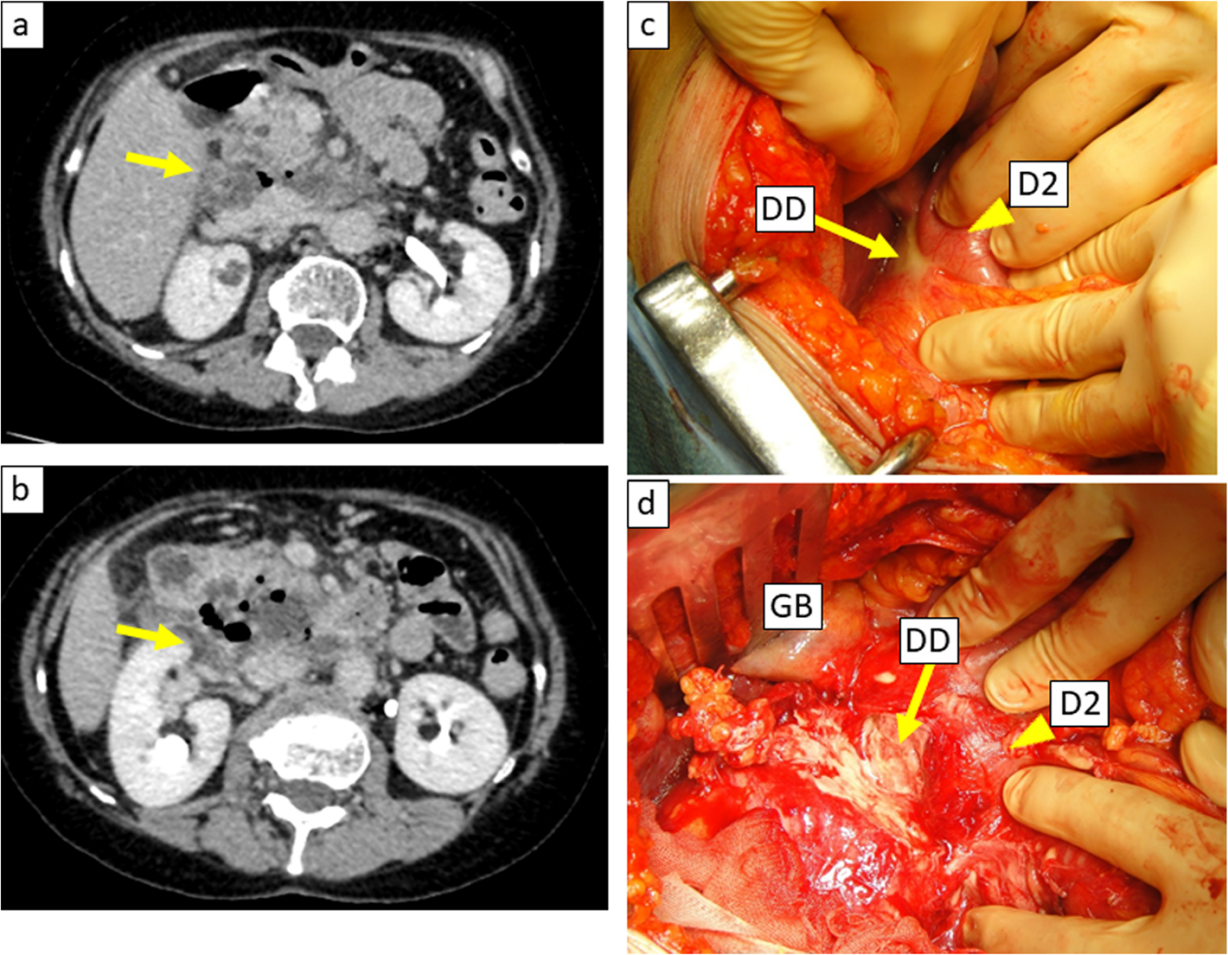
**a**, **b** Computed tomography images showing abscess with free air surrounding the second portion of the duodenum (yellow arrow). **c**, **d** Surgical findings of the perforation site. The perforated duodenal diverticulum (DD, yellow arrow) was identified on the dorsal side of the second portion of the duodenum (D2, yellow arrowhead; GB, gallbladder)

**Fig. 2:**
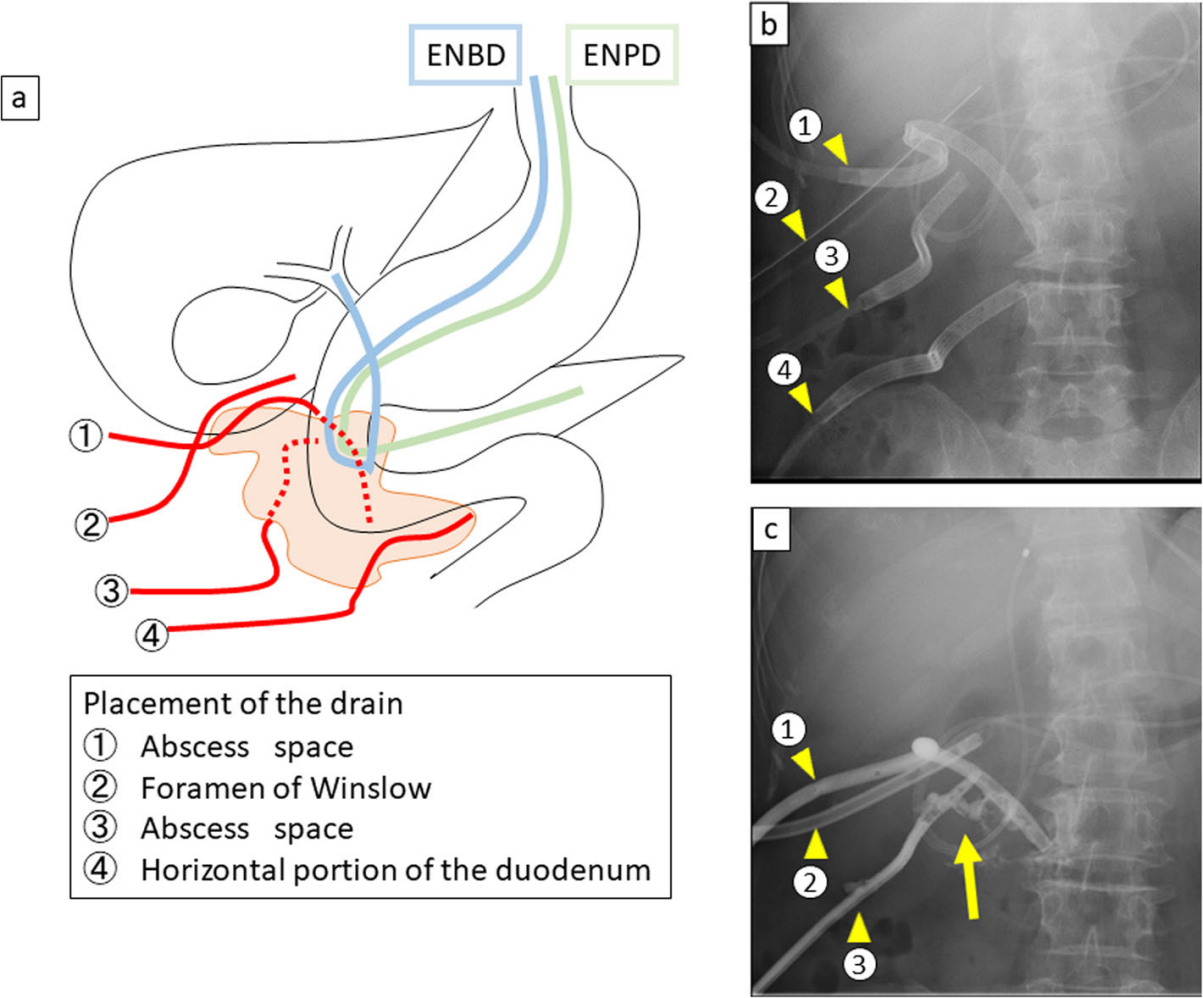
**a** The scheme of the treatment strategy. Four indwelling tubes (① abscess space, ② foramen of Winslow, ③ abscess space, and ④ horizontal part of the duodenum) were placed intraoperatively. Furthermore, endoscopic nasobiliary drainage (ENBD) and nasopancreatic drainage (ENPD) tubes were placed on postoperative day (POD) 2. **b** The picture after endoscopic retrograde cholangiopancreatography (ERCP) on POD 2. Yellow arrowheads show the placement of four indwelling tubes. **c** The picture of contrast radiography on POD 20, which revealed a fistula between the abscess space and the duodenal diverticulum (yellow arrow). The drain placed on the horizontal part of the duodenum was already removed, and yellow arrowheads show that the three indwelling drains remained

**Fig. 3:**
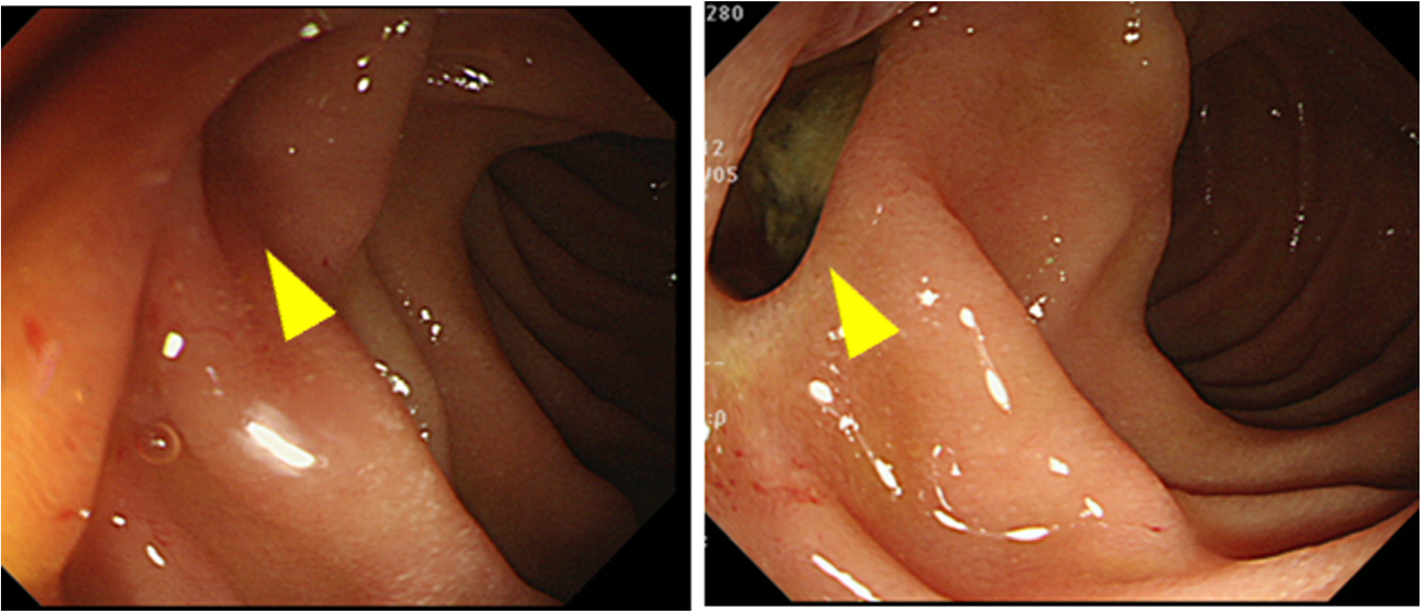
Upper gastrointestinal endoscopy performed 2 months after surgery revealed the presence of food debris in the duodenal diverticulum (yellow arrowhead)

The patient was a 60-year-old female who came to the hospital with chief complaints of fever and right dorsal pain. She had no particular past medical history. She had continuous fever lasting for 1 week and had visited a nearby clinic. Because the laboratory findings revealed the elevation of inflammation markers, she was referred to our hospital for further examination and treatment. Her vital signs showed a body temperature of 37.2 °C and heart rate of 119 beats/min. On physical examination, she complained of abdominal pain in the right upper quadrant of the abdomen with no signs of peritoneal irritation, and right costovertebral angle tenderness was also detected. Laboratory data were remarkable with elevation of inflammatory markers (white blood cell count, 12,900/mm^3^; C-reactive protein, 23.3 mg/ml) and elevation of hepatic bile duct enzymes. Computed tomography (CT) revealed free air with abscess formation surrounding the second portion of the duodenum (Fig. 1). We diagnosed as duodenal perforation with abdominal abscess and decided to perform immediate exploratory laparotomy. During surgery, the perforation site was found to be at the dorsal side of the descending limb of the duodenum. The abscess was formed due to the perforation of a duodenal diverticulum into the retroperitoneum. However, the inflammation surrounding the perforation was too severe, and it was difficult to perform primary closure or dissection of the diverticulum (Fig. 1); therefore, we finished the surgery by placing four intraperitoneal tubes as shown in Fig. 2. The total operation time was 105 min, and the blood loss was 136 ml. Since postoperative day (POD) 1, the leakage of pancreatic juice and bile was continuing (amylase and total bilirubin in the drainage fluid, 105,410 IU/l and 27.4 mg/dl, respectively), and the elevation of inflammation markers appeared to be uncontrollable. In order to manage the leakage of pancreatic juice and bile, we decided to perform endoscopic retrograde cholangiopancreatography on POD 2, and we inserted endoscopic nasobiliary drainage (ENBD) and nasopancreatic drainage (ENPD) tubes. After endoscopic drainage, the amount of drainage decreased, and the drainage fluid became more serous. On POD 6, we began irrigation through the drainage tube in order to continuously wash out the abscess. The contrast radiography on POD 20 revealed a fistula between the abscess and the duodenal diverticulum which gradually decreased in size (Fig. 2). We removed the ENPD tube on POD 34 and the ENBD tube on POD 41. We started oral ingestion on POD 42, and the patient was discharged on POD 57. Upper gastrointestinal endoscopy performed 2 months postoperatively revealed the duodenal diverticulum containing food debris (Fig. 3).

## Discussion

The incidence of duodenal diverticula detected at autopsy is reported to be as high as 22% [10]. Duodenal diverticula are commonly found in the parapapillary of Vater and the horizontal portion and ascending portion of the duodenum. Most diverticula are asymptomatic, and only 5% of patients experience complications such as acute diverticulitis, hemorrhage, perforation, biliary obstruction, and pancreatitis [2, 11]. The causes of perforated duodenal diverticulum can be diverticulitis, ulceration, enterolithiasis, foreign bodies, and blunt abdominal trauma, and diverticulitis is reported to be the most common cause among all [7, 12]. Perforated duodenal diverticulum is a rare but serious complication associated with a significant mortality rate of up to 30% [13]. Yeh reviewed 186 cases of perforated duodenal diverticulum reported in the literature from 1907 to 2016, and the overall mortality was found to be 16% [14].

A clinical presentation of a perforated duodenal diverticulum is highly valuable, but it is difficult to diagnose at first sight. Some patients may complain of back pain, especially if the perforation is retroperitoneal. Other symptoms will be fever, nausea, and vomiting [15]. CT is effective for the diagnosis showing extraluminal gas and extraluminal fluid, and it is the most useful modality in the diagnosis of a perforated duodenal diverticulum [15].

A total of 201 cases of perforated duodenal diverticulum have been reported worldwide since 1907 up to now [3–5, 7, 13–24]. Including our single case, a summary of all 202 cases is provided in Table 1. The most frequently performed treatment for perforated duodenal diverticulum has been diverticulectomy. A total of 17% of all reported cases were treated by conservative therapy. Applicable surgical procedures should be determined based on the severity of inflammation surrounding the perforation site. If the inflammation is too severe and the risk of anastomosis leakage may be unavoidable, we may be forced to dissect the intestine, leading to pancreatoduodenectomy [3–6]. However, the surgical procedure may be highly invasive for the patients. Instead of these invasive treatments, more conservative treatment is drawing our attention. Eeckhout et al. reported the endoscopic approach to the abdominal abscess due to a perforated duodenal diverticulum [8]. To date, there are only few reports of endoscopic treatment selected for perforated duodenal diverticula [15].

**Table 1:** Reported cases of perforated duodenal diverticulum

		*n* = 202	
Age		Mean 64	(5–94)
Gender	Male	69	(34.2%)
	Female	128	(63.3%)
	Not specified	5	(2.5%)
Location	Second portion	159	(78.7%)
	Third portion	26	(12.8%)
	Fourth portion	5	(2.5%)
	Second and third portions	4	(2.0%)
	Not specified	8	(4.0%)
Treatment	Surgical treatment	168	(83.0%)
	Diverticulectomy	67	(33.2%)
	Diverticulectomy + other surgical procedures	45	(22.3%)
	Primary closure	5	(2.5%)
	Duodenojejunostomy/gastrojejunostomy	5	(2.5%)
	Duodenectomy	3	(1.5%)
	Gastrectomy	1	(0.5%)
	Pancreatoduodenectomy	3	(1.5%)
	Drainage/laparotomy	25	(12.5%)
	ENBD/ENPD with surgical drainage	1	(0.5%)
	Not specified	10	(5.0%)
	Conservative treatment	34	(17.0%)
	Bowel rest/antibiotics	17	(8.5%)
	Percutaneous drainage	2	(1.0%)
	Endoscopic abscess drainage	3	(1.5%)
	No surgery/autopsy only	12	(6.0%)

*ENBD* endoscopic nasobiliary drainage, *ENPD* endoscopic nasopancreatic drainage

In the present case, surgical drainage for abdominal abscess was initially performed; however, the inflammation was too severe to safely perform any surgical procedure in addition to the drainage. In order to irrigate the abscess cavity postoperatively, we placed two drainage tubes into the abscess space and finished without performing omentum patch. CT-assisted percutaneous drainage may be the choice which can be the alternative to the surgery; however, the abscess which was formed gradually in 1 week was very large, and we considered that percutaneous drainage alone might be insufficient to manage the abscess. Instead, we decided to perform laparotomy with sufficient drainage and planned for postoperative irrigation to continuously wash out the abscess. We then performed ENBD and ENPD to control the leakage of bile and pancreatic juice. Endoscopic treatment can be selected not only for direct abscess drainage, but also for bile duct and pancreatic duct drainage. We believe that endoscopic treatment may help these patients from the worst situation by controlling the leakage of bile and pancreatic juice. With regard to our case, if we could not successfully perform endoscopic treatment, we were thinking of performing pancreatoduodenectomy. Fortunately, the endoscopic challenge was effective in our case. Consequently, we could avoid performing any further invasive surgery for these patients in addition to the drainage. To the best of our knowledge, our case is the first to show the effectiveness of ENBD and ENPD in combination with surgical drainage for perforated duodenal diverticulum.

When we encounter a perforated duodenal diverticulum, any possible leakage of bile and pancreatic juice that may be difficult to control only by conservative therapy should be carefully assessed. Although we think that ENBD and ENPD may be effective for the treatment of perforated duodenum diverticulum, surgery should be considered at any time if the situation gets worse.

## Conclusions

ENBD and ENPD can be an effective treatment for perforated duodenal diverticulum. However, if we choose conservative treatment for patients with perforated duodenal diverticulum, we should not hesitate to proceed to surgical intervention at any time the situation gets worse.

## Data Availability

All data generated or analyzed during this study are included in this published article.

## Abbreviations

POD: Postoperative day
CT: Computed tomography
ENBD: Endoscopic nasobiliary drainage
ENPD: Endoscopic nasopancreatic drainage
